# Circulating Micro-RNAs as Potential Blood-Based Markers for Early Stage Breast Cancer Detection

**DOI:** 10.1371/journal.pone.0029770

**Published:** 2012-01-05

**Authors:** Michael G. Schrauder, Reiner Strick, Rüdiger Schulz-Wendtland, Pamela L. Strissel, Laura Kahmann, Christian R. Loehberg, Michael P. Lux, Sebastian M. Jud, Arndt Hartmann, Alexander Hein, Christian M. Bayer, Mayada R. Bani, Swetlana Richter, Boris R. Adamietz, Evelyn Wenkel, Claudia Rauh, Matthias W. Beckmann, Peter A. Fasching

**Affiliations:** 1 Department of Obstetrics and Gynaecology, University Hospital Erlangen, Friedrich-Alexander University Erlangen-Nuremberg, Erlangen, Germany; 2 Comprehensive Cancer Center Erlangen-Nuremberg, Friedrich-Alexander University Erlangen-Nuremberg, Erlangen, Germany; 3 Institute of Diagnostic Radiology, University Hospital Erlangen, Erlangen, Germany; 4 Institute of Pathology, University Hospital Erlangen, Erlangen, Germany; 5 Division of Hematology and Oncology, Department of Medicine, David Geffen School of Medicine, University of California Los Angeles, Los Angeles, California, United States of America; Deutsches Krebsforschungszentrum, Germany

## Abstract

**Introduction:**

MicroRNAs (miRNAs, miRs) are a class of small, non-coding RNA molecules with relevance as regulators of gene expression thereby affecting crucial processes in cancer development. MiRNAs offer great potential as biomarkers for cancer detection due to their remarkable stability in blood and their characteristic expression in many different diseases. We investigated whether microarray-based miRNA profiling on whole blood could discriminate between early stage breast cancer patients and healthy controls.

**Methods:**

We performed microarray-based miRNA profiling on whole blood of 48 early stage breast cancer patients at diagnosis along with 57 healthy individuals as controls. This was followed by a real-time semi-quantitative Polymerase Chain Reaction (RT-qPCR) validation in a separate cohort of 24 early stage breast cancer patients from a breast cancer screening unit and 24 age matched controls using two differentially expressed miRNAs (miR-202, miR-718).

**Results:**

Using the significance level of p<0.05, we found that 59 miRNAs were differentially expressed in whole blood of early stage breast cancer patients compared to healthy controls. 13 significantly up-regulated miRNAs and 46 significantly down-regulated miRNAs in our microarray panel of 1100 miRNAs and miRNA star sequences could be detected. A set of 240 miRNAs that was evaluated by radial basis function kernel support vector machines and 10-fold cross validation yielded a specificity of 78.8%, and a sensitivity of 92.5%, as well as an accuracy of 85.6%. Two miRNAs were validated by RT-qPCR in an independent cohort. The relative fold changes of the RT-qPCR validation were in line with the microarray data for both miRNAs, and statistically significant differences in miRNA-expression were found for miR-202.

**Conclusions:**

MiRNA profiling in whole blood has potential as a novel method for early stage breast cancer detection, but there are still challenges that need to be addressed to establish these new biomarkers in clinical use.

## Introduction

Breast cancer (BC) is one of the leading causes of cancer death among women worldwide [1]. A plethora of studies on BC detection and treatment has been published in recent years. Whereas most studies focus on the improvement of prognosis by improving the therapies, one of the most promising approaches is to detect the cancer in an early stage. Mammography and ultrasound are currently the standard diagnostic tools which have been proven to be successful for the detection of early stage BC, but new minimally invasive diagnostic approaches are still urgently needed to supplement breast imaging and to improve detection rates and BC screening compliance.

MicroRNAs (miRNAs, miRs) are a novel class of endogenous, non-coding, single-stranded RNAs, first described in 1993 by Lee et al. in C. elegans [2]. These small regulatory RNA molecules (approximately 22 nucleotides long) post-transcriptionally inhibit gene expression by either degrading or blocking translation of messenger RNA (mRNA) targets [3]. MiRNAs suppress the translation of target mRNAs mainly by binding to their 3′untranslated region (UTR), but also other mechanisms have been described [4]–[7]. Depending on the degree of concordance between the miRNA sequence and the mRNA the negative regulatory effect on the target mRNAs can vary from weak repression of protein translation to complete cleavage of the mRNA [8].

MiRNA loci are statistically over-represented at fragile genomic regions that are commonly amplified or deleted in human cancers, implying a connection of miRNAs with cancer initiation and progression [9]–[11]. Whether miRNAs act mainly as tumor suppressors (suppressor-miRs), promotors of tumorigenesis (onco-miRs) or both is still widely elusive, but the global decrease in miRNA expression in human cancers suggests that most miRNAs may act as direct suppressor-miRs or post-transcriptional repressors of known oncogenes [12]–[17].

The finding of a decrease of miR-15a and miR-16-1 in patients with chronic lymphocytic leukaemia was one of the first direct links between regulative miRNAs and cancer [18]. Meanwhile, miRNAs have been implicated in nearly all human cancers and especially the relevance in BC has been shown by several groups [19]–[23]. Some miRNAs were found to be up-regulated in BC tissue compared to normal breast tissue and other miRNAs were down-regulated, which is in accordance with the hypothesis of miRNAs acting as onco-miRs and tumor-suppressor miRs [19]. After the first identification of serum miRNAs in 2008 [24], several studies have shown that miRNAs are present in fluids like blood, saliva, pleural fluid and urine [25]–[28]. The extreme stability of circulating miRNAs in the RNase-rich environment of the bloodstream is the basis of their value as biomarkers, but the mechanism underlying this stability is to a large extent still elusive. Initial studies indicated that miRNAs are protected from degradation by inclusion in lipid or lipoprotein complexes like microvesicles, exosomes, or apoptotic bodies [23], [29], [30]. Surprising results from a very recent study revealed that the majority of circulating miRNAs co-fractionate with protein complexes and could be protected in the circulation by these complexes [31]. The identification of circulating miRNAs as potential non-invasive biomarkers for BC and other diseases was followed by initial studies trying to associate these markers with relapse-free survival, overall survival and response to therapy [23]–[26], [32]–[38].

Aim of the present study was to analyze the miRNA expression patterns in whole blood of patients with early stage BC in comparison to healthy controls using a miRNA microarray chip.

## Methods

All patients of this study participated in a prospective case control study for the molecular detection of breast cancer (MODE-B Study). Patients presenting at our specialized breast cancer unit with suspect breast lesions are routinely asked to participate in this still ongoing MODE-B Study. The presented data are results from the monocentric miRNA pilot-study.

### Ethics Statement

The MODE-B Study was approved by the Ethics Committee of the Medical Faculty of the Friedrich-Alexander University Erlangen-Nuremberg (reference number 3937). Written informed consent was obtained from every patient and control individual before blood was taken.

### Samples

Venous blood samples (non-fasting) (2.7 mL per patient) were collected from cases and controls in EDTA blood tubes (Sarstedt, Monovette EDTA K; Sarstedt AG, Germany) containing 1.6 mg EDTA as anticoagulant and stored at −20°C until further processing. We selected the first 48 consecutive early stage BC patients suitable for this prospectively planed miRNA-biomarker-study.

### Patient characteristics

This study comprises two case control studies, a discovery study (microarray chip analysis) and a validation study (RT-qPCR of selected miRNAs). Patients were included in the specialized breast unit. They either referred themselves because of a newly palpable breast lesion or were referred to the breast unit by their physicians because of suspicious lesions for further breast diagnostics and biopsy.

The independent RT-qPCR validation cohort consisted of consecutive early stage BC patients diagnosed within the German BC screening program. These differences in patients' recruitment between the microarray study cohort and the RT-qPCR validation cohort resulted inevitably in a lower risk profile of patients in the validation cohort (Table 1), which was intended as ultimate test of the discriminating potential of RT-qPCR based miRNA-profiling. The control cohort included individuals with inconspicuous mammograms who came to our hospital for a routine check and had no history of current or previous malignancy. All patients and controls were of Western European descent. The demographic and clinicopathological patients' characteristics of all BC patients in the microarray cohort and in the RT-qPCR validation cohort are summarized in Table 1. Tumor staging was done according to the tumor-node-metastasis (TNM) staging system of the American joint committee on cancer (AJCC) and the International Union for Cancer Control [39].

**Table 1:** Patients' and tumor characteristics at time of BC diagnosis.

Parameters	Microarray cohort patients (%)	RT-qPCR validation cohort patients (%)
Total	48	24
Age	61.9 (range 34–89 years)	age matched pairs
**Tumor stage**		
pT1a	0	3 (12.5)
pT1b	12 (25.0)	6 (25.0)
pT1c	29 (60.4)	11 (46.0)
pT2	7 (14.6)	4 (16.5)
**Lymph node involvement**		
pN0	39 (81.25)	23 (96.0)
pN1 (1–3 lymph nodes)	9 (18.75)	1 (4.0)
**Grading**		
G1	12 (25.0)	10 (42.0)
G2	19 (39.6)	12 (50.0)
G3	17 (35.4)	2 (8.0)
**Estrogen receptor status**		
Positive	44 (91.7)	21 (87.5)
Negative	4 (8.3)	3 (12.5)
**Progesterone receptor status**		
Positive	39 (81.25)	16 (67.0)
Negative	9 (18.75)	8 (33.0)
**Her2 status**		
Positive	40 (83.3)	1 (4.0)
Negative	8 (16.7)	23 (96.0)
**Ki-67**		
**<10**	11 (22.9)	4 (16.7)
10	13 (27.1)	11 (45.8)
10–20	10 (20.9)	6 (25.0)
>20	14 (29.1)	3 (12.5)

### MiRNA extraction and miRNA-microarray profiling

Total RNA extraction was performed as published previously [40]. After unfreezing, EDTA blood was transferred into PAXgene Blood RNA Tubes (PreAnalytiX GmbH, Switzerland) as described previously, and total RNA was extracted using the miRNeasy kit (Qiagen GmbH, Hilden, Germany) with minor modifications. RNA was eluted in water and shipped on dry ice to be analysed on febit's Geniom® real-time analyser (GRTA, febit gmbh, Heidelberg, Germany) using the Geniom® Biochip miRNA homo sapiens

The quality and quantity of the RNA was evaluated by 260/280 ratio using NanoDrop spectrophotometry (NanoDrop ND-1000) and Agilent 2100 Bioanalyzer (Agilent Technologies Inc., Santa Clara, CA).

Each array of the Geniom® Biochip contains 11 replicates of 1100 miRNAs and miRNA star sequences as annotated in the Sanger database miRBase15.0 [41]. Samples were biotinylated using microfluidic-based enzymatic on-chip labeling of miRNAs (MPEA) [42]. After hybridisation for 16 h at 42°C, the biochip was washed automatically and a program for signal enhancement was processed with the GRTA. Results were analysed using the Geniom® Wizard Software. For each array, the median signal intensity was extracted from the raw data file such that for each miRNA seven intensity values have been calculated corresponding to each replicate copy of miRNA-Base on the array. After background correction, median values were calculated from the seven replicate intensity values of each miRNA. To normalise arrays, variance stabilising normalisation (VSN) as implemented in the R package VSN has been applied and all further analyses were carried out using the normalised and background-subtracted intensity values [43].

### MiRNA MicroArray Data Analysis

The approximate normal distribution of the measured data was verified by Shapiro–Wilk test with a median P-value of 5.9E-20. MiRNAs with different expression levels between BC patients and controls were identified by unpaired two-tailed parametric t-test. P-values obtained for each individual miRNA were adjusted for multiple testing by Benjamini–Hochberg adjustment [44]. In addition to the single biomarker analysis, samples were also classified according to miRNA patterns as calculated using support vector machines (SVMs) implemented in the R (Team, 2008) e1071 package [45]. In detail, different kernel (linear, polynomial, sigmoid, radial basis function) SVMs were evaluated with the cost parameter being sampled from 0.01 to 10 in decimal powers. The measured miRNA profiles were classified using 100 repetitions of standard 10-fold cross-validation and subsets were selected according to a t-test-based filter approach. This means that in each repeat of the cross-validation the “s” miRNAs with lowest P-values were computed on the training set with “s” being sampled according to the included number of miRNAs [46]. The respective subset was then used to train the SVM for the prediction of the test samples, which enabled a calculation of the mean accuracy, specificity and sensitivity for each subset size. Permutation tests were applied to check for overtraining. In this study, the class labels were sampled at random and classifications were carried out using the permuted class labels [46]. The expression profile of each differentially expressed miRNA was used for the creation of Receiver Operating Characteristic (ROC) curves, a graphical plot of the true positive rate versus the false positive rate. The area under the ROC curve (AUC) is representing the discrimination accuracy and is shown in Table 2 and 3 for the most deregulated miRNAs found by microarray profiling. All statistical analyses were performed using R (Wilcoxon, 1945; R development Core Team, 2008). The microarray data has been submitted to a public repository (Gene Expression Omnibus, GEO) and has been approved and assigned GEO accession numbers (GSE3109, GPL14132).

**Table 2:** List of the ten most up-regulated miRNAs in BC patients detected by highest absolute value of fold changes.

miRNAs	Median controls	Median BC pat.	Fold change	T-test raw P-value	T-test adj P-value	AUC
miR-4306	10.09	10.83	2.08	0.0007	0.02	0.71
miR-202	4.49	5.21	2.04	0.0004	0.02	0.72
miR-4257	5.50	6.17	1.96	0.0017	0.04	0.65
miR-1323	5.48	6.13	1.92	0.0022	0.04	0.69
miR-335	7.52	8.16	1.89	0.0017	0.04	0.74
miR-497	7.28	7.87	1.82	6.56E-05	0.01	0.75
miR-106b	13.22	13.76	1.69	0.0004	0.01	0.72
miR-922	6.98	7.49	1.67	0.0019	0.03	0.65
miR-516b	5.59	6.09	1.64	0.0008	0.03	0.67
let7a*	5.10	5.40	1.35	0.001	0.03	0.65

BC pat. … breast cancer patients; adj … adjusted.

**Table 3:** List of the fifteen most down-regulated miRNAs in BC patients detected by highest absolute value of fold changes.

miRNAs	Median controls	Median BC pat.	Fold change	T-test raw P-value	T-test adj P-value	AUC
miR-718	7.26	6.12	3.12	4.27E-05	0.0041	0.77
miR-625*	7.57	6.46	3.05	1.19E-05	0.0020	0.77
miR-1471	6.41	5.61	2.24	0.0002	0.0127	0.70
miR-193a-3p	7.34	6.57	2.16	1.67E-07	0.0001	0.79
miR-182	12.67	11.91	2.14	0.0001	0.0080	0.71
miR-1915	8.73	8.05	1.98	1.53E-05	0.0022	0.75
miR-564	7.26	6.60	1.93	0.0003	0.0127	0.67
miR-107	12.41	11.80	1.83	0.00193	0.0414	0.68
miR-2355	7.15	6.55	1.82	4.91E-05	0.0042	0.73
miR-3186-3p	6.96	6.37	1.81	6.39E-06	0.0015	0.75
miR-24	11.54	10.94	1.81	0.0007	0.0235	0.65
miR-3130-3p	7.89	7.29	1.81	5.03E-05	0.0042	0.73
miR-526a	7.33	6.77	1.76	0.0003	0.0127	0.72
miR-1469	7.17	6.64	1.70	0.0001	0.0080	0.68
miR-874	7.53	7.01	1.67	2.10E-06	0.0006	0.74

BC pat. … breast cancer patients; adj … adjusted.

### Validation of miRNA gene expression using real-time semi-quantitative PCR (RT-qPCR)

Two miRNA (miR-202, miR-718) which were differentially expressed in the microarray assays were analyzed in an independent validation cohort by RT-qPCR. Among the most deregulated miRNAs from our microarray experiments we selected miRNAs for PCR-validation with purchasable and well established primers to reduce sources of error. The miRNA isolation and RNA quantification of the independent validation cohort was identical to the microarray analysis. TaqMan miRNA assays from ABI (Applied Biosystems, Foster City, CA, USA) were purchased for miR-202 and miR-718. First, each miRNA was specifically reverse transcribed according to manufactures protocol using TaqMan miRNA RT-Kit with stem-loop RT-primer and ABI7300 (ABI). Second, each sample was analyzed in duplicates for each specific miRNA using a RT-primer with universal master mix II (without Uracil-N-glycosylase) on the Applied Biosystems 7300 Sequence Detection System according to manufactures protocol. The cycle thresholds (Ct) for BC patients and their age-matched healthy controls were calculated and normalized to miR-16 (miR-16; ABI), which was found in the literature as the most widely-used endogenous control miRNA for RT-qPCR. Each analysis also contained inter- and intra-assay replicates. High Ct-values indicated low miRNA quantity and vice versa. The expression levels of miRNAs in BC patients relative to their age-matched healthy controls were calculated using the comparative cycle threshold (*C*_T_) method. The average *C*_T_ value of the control miR-16 for every sample was subtracted from the *C*_T_ value for each respective mature miRNA reaction, resulting in the Δ*C*_T_ value. The fold changes in miRNAs were calculated by the equation 2^−ΔΔCt^ where the comparative cycle threshold (ΔΔ*C*_T_) is defined as the difference between Δ*C*_T_ (Cancer) minus Δ*C*_T_ (Control) as described previously [23], [47], [48]. Differences between miRNA expression levels among two groups were evaluated using the t-test. We set the alpha-level to consider miRNAs as significant to 0.05. The expression profile of miR-202 in the RT-qPCR validation was used for the creation of a ROC curve.

## Results

### Characterization of study population

In total, 153 whole blood samples of early stage BC patients and healthy control individuals were analyzed in this study. Clinical patient and tumor characteristics at time of BC diagnosis are shown in Table 1. The cases and controls were age-matched in the RT-qPCR validation cohort. Age-matching was not performed in the discovery part of our study, but cases and controls in the microarray cohort were in the same age group (mean age: 62y vs 58y). All BC cases were histologically confirmed as early stage invasive ductal carcinoma of the breast with a tumor size ranging between 0.15 and 4.0 cm. Hormone receptor status (estrogen/progesterone receptors (ER/PR)), Ki-67 status, grading and HER2-status were available for all patients (Table 1).

### Discovery of differentially expressed miRNAs in blood samples of BC patients by miRNA microarray profiling

**Figure 1:**
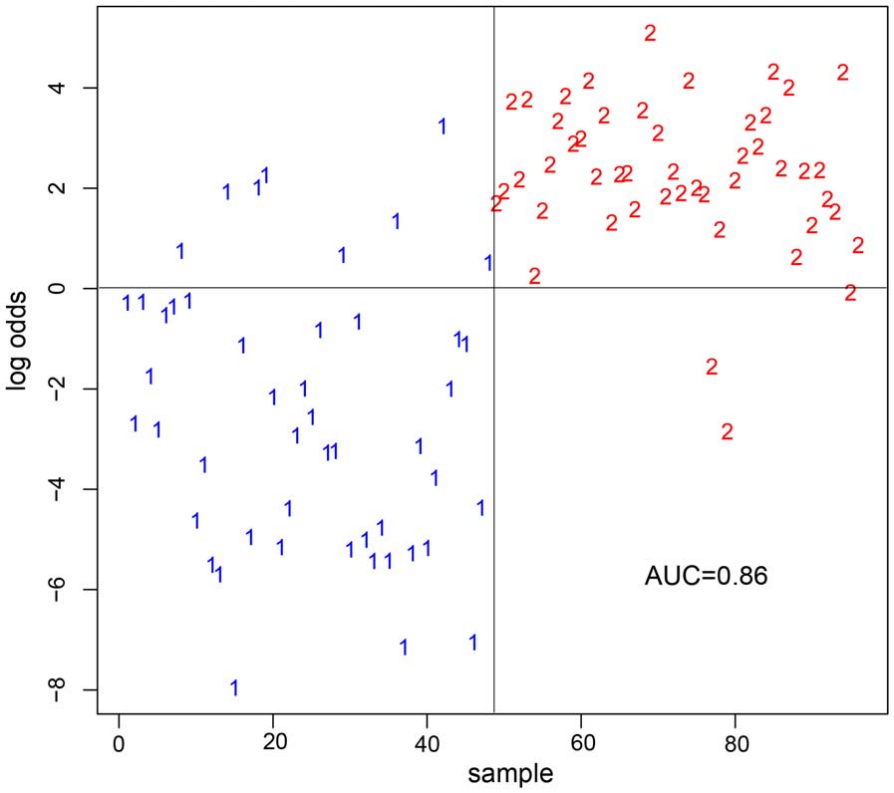
Representative example of a classification result using trained support vector machines. The graph shows the logarithm of the quotient of the probability to be BC sample and the probability to be a control sample (log odds) of all samples analyzed with miRNA-microarrays. 1… Controls; 2…BC patient.

In the discovery setting, miRNA-microarray analyses of 48 BC cases using the Geniom® Realtime Analyzer microarray platform identified 59 deregulated miRNAs in whole blood of early stage BC patients compared to healthy controls [49]. T-test with Benjamini–Hochberg adjustment revealed 13 significantly up-regulated miRNAs and 46 significantly down-regulated miRNAs in our microarray panel of 1100 miRNAs and miRNA star sequences. We also tested different subsets of the most deregulated miRNAs to discriminate cancer cases from controls (Figure S1). Sensitivity, specificity and accuracy increases continuously with increasing numbers of miRNAs used for discrimination of cancer cases and controls. Using a subset of 30 miRNAs the values of all three variables were over 80 percent, while we were able to reach the optimal classification results, with an accuracy of 85.6%, a specificity of 78.8%, and a sensitivity of 92.5% with a subset of 240 miRNAs, indicating that besides the 59 statistically significant deregulated miRNAs, other miRNAs may also contain diagnostic information that could improve the classification result. An example of a classification result using these 240 miRNAs is shown in Figure 1. Table S1 shows miRNAs with significant expression differences between distinct tumor subgroups. The most deregulated 25 miRNAs detected by highest absolute value of logarithmized fold changes between BC patients and healthy controls are shown in Table 2 (up-regulated miRNAs in BC) and Table 3 (down-regulated miRNAs in BC).

### Results of RT-qPCR validation

The expression levels of two miRNAs with purchasable and well established primers were confirmed with a Taqman-based RT-qPCR in an independent cohort of BC patients and controls using individual miRNA-specific primers. MiR-202 which showed up-regulation pattern for BC cases over controls in the microarray analyzes and miR-718 which was identified with down-regulation pattern were selected to be validated in an independent cohort of 24 age matched pairs of early stage BC patients and 24 healthy controls. We were able to validate the microarray separation pattern of the two miRNAs in the validation cohort using RT-qPCR. The PCR results are summarized in Table 4.

**Table 4:** Comparison of miRNA expression fold changes between microarray and RT-qPCR.

miRNA	Relative fold change RT-qPCR	T-test P-value RT-qPCR validation	Changes in BC cases	Fold change microarray
miR-202	19.38	0.03	Up-regulation	2.04
miR-718	5.44	0.72	Down-regulation	3.12

**Figure 2:**
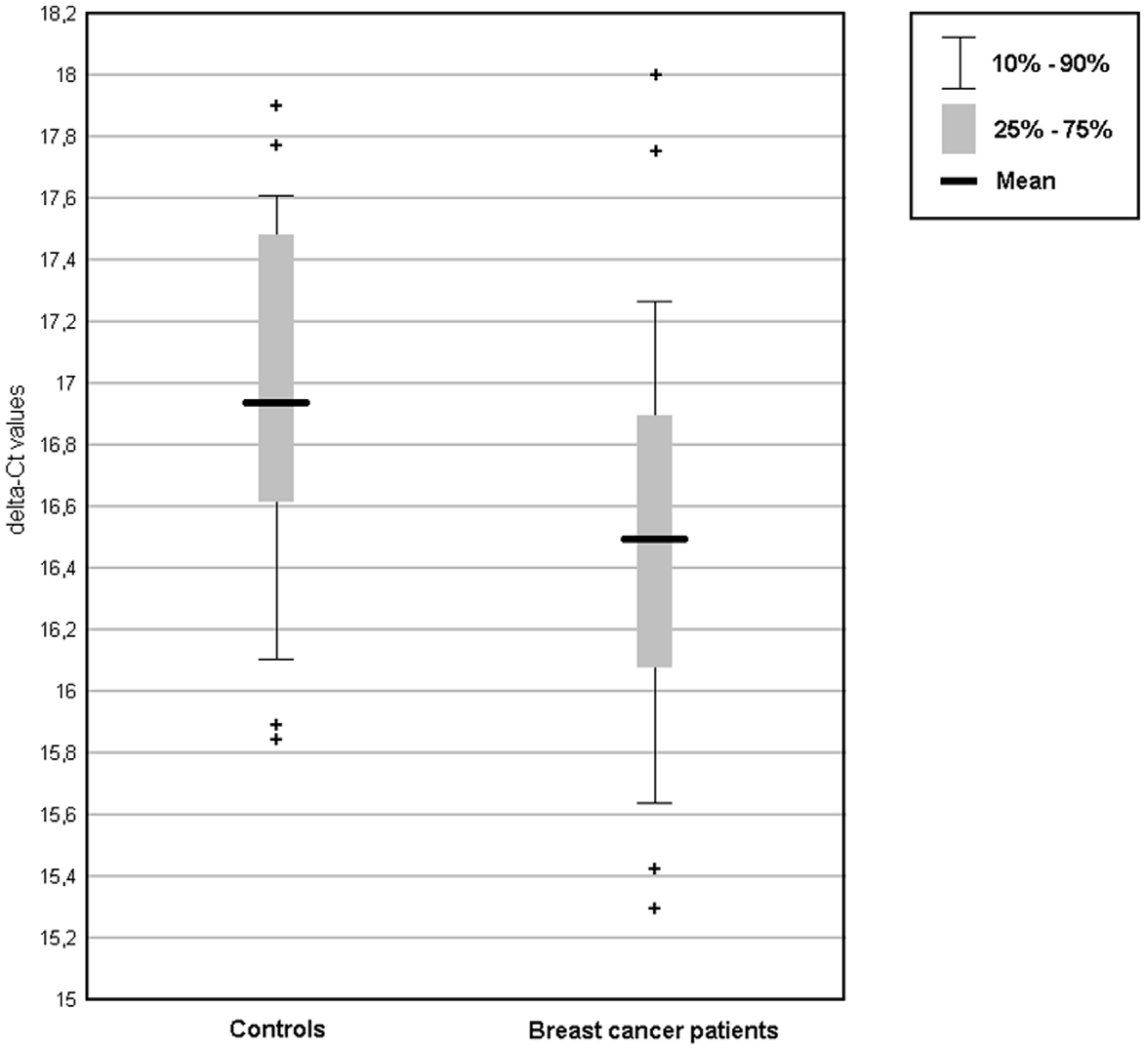
RT-qPCR validation of miR-202. Significant different expression of circulating miR-202 in whole blood of BC patients versus Controls (up-regulation of miR-202). Data derived from RT-qPCR and presented as delta-Ct values, with higher values standing for lower miRNA-expression.

**Figure 3:**
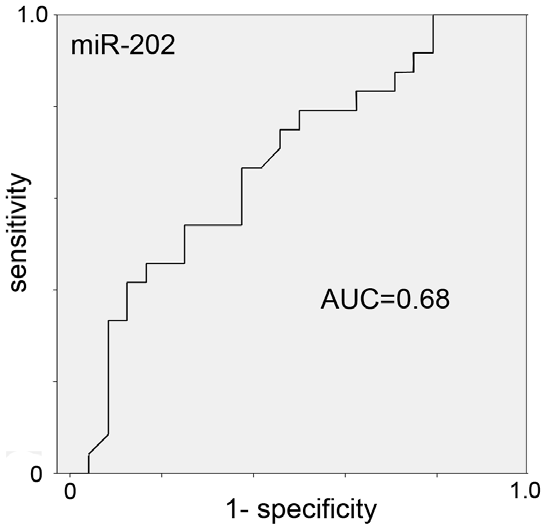
Area under the receiver operating characteristic curve (AUC) for miR-202 based on the RT-qPCR data.

RT-qPCR results were concordant with the miRNA microarray results in terms of up- and down-regulation calculated as relative fold changes in comparison with each age matched control and calculated as relative fold changes between the two groups (Table 4). A discrimination between the two groups using RT-qPCR expression values of a single miRNA was only possible for miR-202, but not miR-718 in our small validation cohort (Figure 2 and 3).

## Discussion

Mammography is currently the modality of choice for screening for early BC and possesses a sufficient sensitivity and specificity. Specificity of screening mammography is over 95%, but sensitivity ranges between 67% and 95% and is strongly dependent on several factors like age, breast density and professional experience of the examiner [50]–[52]. In women with very dense breast tissue sensitivity of screening mammograms can be as low as 40 to 50% [52]. Magnetic resonance imaging (MRI) of the breast and breast ultrasound could improve cancer detection in these cases, but these imaging techniques are not routinely used for BC screening [52], [53]. Therefore, intensive research is currently carried out to identify new, non-invasive BC detection methods. In the discovery setting of our study we found 13 significantly up-regulated miRNAs and 46 significantly down-regulated miRNAs in our microarray panel of 1100 miRNAs and miRNA star sequences and validated our results in an independent BC cohort with RT-qPCR. A set of 240 miRNAs yielded a specificity of 78.8%, and a sensitivity of 92.5% in this very early stage BC cohort. We deliberately used frozen EDTA-blood to test and establish a miRNA-detection method which could possibly be used in larger multicentric trials collecting frozen whole blood. The stability of miRNAs in EDTA-blood and the possibility of miRNA-profiling from non-frozen EDTA-blood have been shown previously by others [54]. After miRNA-microarray-profiling miR-202, and miR-718 were chosen for RT-qPCR validation based on the significant expression changes on the microarray. Moreover, the identification of biochemical pathways that are enriched with respect to the miRNA target genes revealed miR-202 as the miRNA with the highest number of significant biological pathways and Gene Ontology categories of all known miRNAs [55]. This target pathway analysis indicates that miR-202, which we found to be significantly up-regulated in our early stage BC blood samples of the microarray and the RT-qPCR cohort, is influencing a plethora of cancer relevant biological pathways and might be important for BC development. Considering that miR-202 belongs to the let-7 family of miRNAs, which are known to be involved in self-renewal and tumorigenicity of BC cells, functional data are available regarding the role of miR-202 and other members of this familiy in carcinogenesis [56], [57]. The let-7 family regulates estrogen receptor alpha signaling in estrogen receptor positive BC and is highly conserved across species in sequence and function [56], [58]. The miRNAs let-7e and miR-202 target the same seed sequence and a recent study has shown that these miRNAs target the proto-oncogene MYCN in vitro [59]. Further targets of the let-7 family of miRNAs are RAS and HMG A2 (high mobility group A2) [60]. The two associated miRNA families, let-7 and miR-200, have also been identified as regulators of epithelial-to-mesenchymal transition (EMT) and dedifferentiation of cancer cells to stem cells [60].

Several miRNAs are known to be aberrantly expressed in human BC tissue and have been correlated with clinical stage and clinico-pathological variables like hormone receptor status, tumor subtypes, as well as clinical variables like metastatic potential, progression free survival and overall survival [19], [61]–[65]. Tissue-based miRNA expression profiling of the inflammatory breast cancer (IBC) subtype observed some miRNAs to be independently associated with the difference between IBC and non-IBC. Among those miRNAs with increased expression in the IBC subtype was also miR-335, which was also significantly up-regulated in our blood-based microarray discovery study [66]. The expression of miR-335 was also up-regulated (3.9 fold) in colonic cancer tissues compared to para-cancerous control tissue [67]. On the other hand, miR-335 has also been reported as robust inhibitor of tumor reinitiation and was found to suppress migration, invasion, and metastatic colonization in vivo [68]. Another study analyzing tissues from malignant pleural mesothelioma found miR-193-3p to be over expressed in formalin-fixed, paraffin-embedded malignant pleural mesothelioma tissue compared to carcinoma tissue [69]. In line with these findings, our microarray study identified miR-193-3p as one of the most significantly down-regulated miRNAs in whole blood of BC patients (2.16-fold; P = 0.0001).

Blood-based miRNA-profiling is still far behind the improvements in tissue-based miRNA-profiling, but offers the potential for early, non-invasive, sensitive and specific BC detection and screening. First reports using serum or plasma for RT-PCR or microarray based miRNA-profiling were promising. Recently, a serum based study using next-generation sequencing of miRNAs for BC detection has also been reported and several miRNAs have been identified as potential serum/plasma biomarkers in different cancer types like lung, prostate, colon and liver cancer [70]–[72]. Furthermore, strategies with whole blood have been established and likewise show favourable results in the non-invasive detection of cancer and other diseases [73]–[76].

One potential advantage of the whole blood approach could be the higher miRNA-content and the chance to measure not only tumor secreted oncogenic miRNAs, but also the changes in the miRNA profile following the “host-reaction” in the body of the patient [77]. On the other hand, the main concern about using whole blood is a reduction of the testing accuracy due to the measurement of a miRNA profile which represents only an unspecific secondary response of blood cells during tumorigenesis [78]. Secondly, the high protein content of whole blood could be a problem for RNA-extraction. Results from previous studies of our and other groups indicated that the changes in the miRNA profile of blood cells of patients with cancer also reflect tumor-specific host-reactions which might be measurable in whole blood [75], [76]. We would therefore expect that the whole blood approach offers the potential to diagnose cancer at a very early stage when the concentration of tumor-secreted miRNAs is still small, but the reaction of the immune system in response to cancer can already be detected by miRNA profiling in whole blood. Comparing serum and blood cells from the same healthy individual an almost identical miRNA profile can be found, but in cancer patients the profiles differ [26]. In contrast to miRNA-studies using plasma or serum we found a contrary trend in whole blood regarding the previous published plasma levels of let-7d*, let-7c, miR-425* and miR-589 [74]. Although none of these miRNAs was significantly differentially expressed in our microarray analyses the median fold changes between BC cases and controls were completely contrary to the plasma expression changes published by Zhao et al. for these four miRNAs in Caucasian Americans (n = 15) [74]. For example, the whole blood expression levels of let-7d* in our microarray cohort (n = 48) were almost 2-fold higher in BC cases compared to controls with a P-value of 0.009 in the unadjusted t-test and 0.098 in the adjusted t-test.

The comparison of miRNA-profiles from whole blood and plasma/serum is also addressed in the work of Zhao et al. [74]. The authors of this plasma based miRNA-profiling study in BC were not able to reproduce the data from Heneghan et al. showing a significantly higher expression of let-7a and miR-195 in whole blood of BC cases compared to controls [74], [79]. In our study we found only a trend towards an up-regulation of miR-195 in whole blood of BC cases compared to controls in the miRNA-microarray analyses (P = 0.055). Possible reasons for this discrepance are differences in sample handling, detection methods and patient selection (clinical stages). Moreover, a recent study showed significant differences between cell-free and cellular blood miRNA profiles. Using different plasma fractionation procedures for plasma, the authors showed varying degrees of efficacy in the removal of red and white blood cells and as a consequence different miRNA profiles [80]. In addition to different detection techniques, these differences could be partially responsible for the discrepancies between blood based miRNA profiling studies. Based on this data whole blood miRNA profiling seems reasonable to achieve integrated, standardized analyses of circulating disease specific miRNA signatures; and is able to measure the disease specific over-expression of hematopoietically derived miRNAs and circulating cell-free miRNAs.

The median fold expression changes of deregulated miRNAs in our microarray analyses were in the range of 2 to 4 fold which is in line with previous miRNA microarray profiling studies, but fare less than previously reported in qRT-PCR based studies. These differences were expected and are probably due to the different detection and analysis techniques. The relative fold changes found in our RT-qPCR validation analyses were higher, with miR-202 and miR-718 showing a relative fold change of about 20 and over 5 between cases and age-matched controls (Table 4). The RT-qPCR validation cohort was small with only 24 age matched pairs and we were able to show statistically significant differences in expression values only for one of the two analysed miRNAs (miR-202), but in agreement with the microarray results the relative fold changes showed the same trend for both miRNAs. This is probably due to the small sample size, but could also indicate that a set of miRNAs rather than a single miRNA is needed for a reliable differentiation of cancer cases and controls.

The clinicopathological characteristics of the two cohorts are slightly different with smaller and less aggressive tumors with a higher rate of HER2-negativity in the RT-qPCR cohort compared to the microarray cohort. This is due to the different recruitment strategy (hospital based versus screening cohort) and the consecutive patient recruitment.

We used miRNA microarray technology to analyze the miRNA expression profiles of early stage BC patients compared to healthy controls from frozen EDTA-whole-blood and validated the results with RT-qPCR in an independent early stage BC cohort. Research of circulating miRNAs as blood based biomarkers is still in its infancy. However, this study as well as other recent studies indicate that miRNA-analyses have diagnostic and prognostic potential and could improve early stage BC detection in the future. There are several possible applications of miRNA profiling conceivable in the future. Firstly, miRNA profiles could help to reduce unnecessary breast biopsies if miRNA sets could be identified which reliably identify BC free individuals. Secondly, miRNA profiling could be used as a pre-screening method for example by general practitioners to identify women with an urgent need for breast diagnostics. Thirdly, in younger patients with dense breast tissue a future miRNA-based BC screening could possibly provide better sensitivity and specificity than the mammography even without radiation exposure.

Our study detected several significant deregulated miRNAs in frozen whole blood of early stage BC patients, which should be analyzed further with regard to their function in breast cancer development and progression. Moreover, large prospective clinical studies are clearly warranted to confirm our preliminary results and further explore the existing potential of circulating miRNAs in serum, plasma or whole blood as diagnostic and therapeutic BC biomarkers.

## Supporting Information

Figure S1 **Classification plot of microarray signatures.** This is a classification plot demonstrating that a multimarker signature increases test accuracy, specificity and sensitivity depending upon the number of miRNAs that compose the diagnostic signature.
(TIF)

Table S1 **MiRNAs differentially expressed in different subgroups.**
(DOC)
