# Supplementary figures and images for: Circulating Micro-RNAs as Potential Blood-Based Markers for Early Stage Breast Cancer Detection
Source: PLoS One. 2012 Jan 5;7(1):e29770. doi: 10.1371/journal.pone.0029770 (PMC3252341; doi:10.1371/journal.pone.0029770)

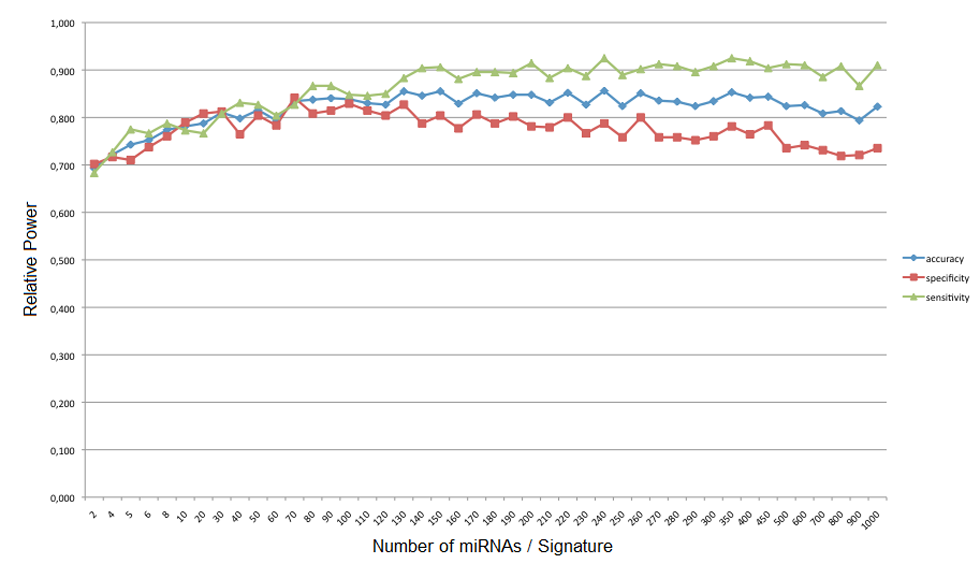

Supplement: Figure S1 — Classification plot of microarray signatures. This is a classification plot demonstrating that a multimarker signature increases test accuracy, specificity and sensitivity depending upon the number of miRNAs that compose the diagnostic signature. (TIF) [file pone.0029770.s001.tif]
