# Supplementary material for: Circulating Micro-RNAs as Potential Blood-Based Markers for Early Stage Breast Cancer Detection
Source: PLoS One. 2012 Jan 5;7(1):e29770. doi: 10.1371/journal.pone.0029770 (PMC3252341; doi:10.1371/journal.pone.0029770)
Supplement: Table S1 — MiRNAs differentially expressed in different subgroups. (DOC) [file pone.0029770.s002.doc]

Table S1 MiRNAs differentially expressed in different subgroups

| **miRNAs** | **MedianER20-39** | **Median ER ≥40** | **Fold change** | **T-test raw P‑value** | **T-test adj P‑value** | **AUC** |
| --- | --- | --- | --- | --- | --- | --- |
| miR-4267 | 6.04 | 6.71 | 0.51 | 7.81E-06 | 0.004 | 0.87 |
| miR-886-5p | 5.49 | 6.21 | 0.48 | 8.43E-06 | 0.004 | 0.86 |
| miR-1274a | 5.55 | 6.35 | 0.45 | 1.04E-05 | 0.004 | 0.82 |
| miR-429 | 4.97 | 5.94 | 0.38 | 2.29E-05 | 0.007 | 0.89 |
| miR-9* | 6.40 | 7.10 | 0.50 | 3.49E-05 | 0.007 | 0.86 |
| miR-302c* | 4.22 | 4.85 | 0.53 | 3.75E-05 | 0.007 | 0.87 |
| miR-329 | 6.92 | 5.99 | 2.54 | 9.58E-05 | 0.016 | 0.11 |
| miR-374b* | 4.56 | 5.01 | 0.64 | 0.0002 | 0.027 | 0.78 |
| miR-618 | 4.67 | 5.14 | 0.62 | 0.0002 | 0.027 | 0.74 |
| miR-561 | 5.33 | 5.99 | 0.52 | 0.0002 | 0.027 | 0.87 |
| miR-30c-2* | 5.54 | 6.00 | 0.63 | 0.0004 | 0.035 | 0.81 |
| miR-200a* | 4.66 | 5.66 | 0.37 | 0.0003 | 0.035 | 0.78 |
| miR-138 | 4.22 | 5.49 | 0.28 | 0.0005 | 0.041 | 0.80 |
| **miRNAs** | **Median Her2 pos** | **Median Her2 neg** | **Fold change** | **T-test raw P‑value** | **T-test adj P‑value** | **AUC** |
| miR-614 | 5.76 | 6.21 | 0.64 | 3.71E-05 | 0.04 | 0.85 |
| **miRNAs** | **Median pT1** | **Median pT2** | **Fold change** | **T-test raw P‑value** | **T-test adj P‑value** | **AUC** |
| miR-1245 | 5.32 | 4.27 | 2.87 | 2.65E-06 | 0.003 | 0.14 |
| miR-1197 | 5.54 | 4.55 | 2.67 | 1.00E-05 | 0.006 | 0.13 |
| miR-369-5p | 5.75 | 4.67 | 2.95 | 3.00E-05 | 0.007 | 0.17 |
| miR-191* | 6.61 | 5.20 | 4.09 | 3.54E-05 | 0.007 | 0.15 |
| miR-523 | 6.12 | 5.49 | 1.87 | 2.13E-05 | 0.007 | 0.15 |
| miR-488* | 6.52 | 5.49 | 2.81 | 2.92E-05 | 0.007 | 0.17 |
| miR-541* | 4.69 | 3.84 | 2.35 | 0.0001 | 0.020 | 0.13 |
| miR-200b | 6.03 | 5.53 | 1.66 | 0.0001 | 0.020 | 0.21 |
| miR-510 | 6.11 | 5.76 | 1.43 | 0.0002 | 0.021 | 0.28 |
| miR-3171 | 5.51 | 4.74 | 2.16 | 0.0002 | 0.021 | 0.19 |
| miR-579 | 5.04 | 4.67 | 1.45 | 2.45E-04 | 0.026 | 0.18 |
| miR-513b | 5.30 | 4.58 | 2.06 | 0.0003 | 0.026 | 0.15 |
| miR-1298 | 5.05 | 4.00 | 2.85 | 0.0004 | 0.030 | 0.17 |
| miR-558 | 6.76 | 5.94 | 2.27 | 0.0003 | 0.030 | 0.17 |
| miR-135b* | 5.92 | 5.05 | 2.37 | 0.0006 | 0.050 | 0.24 |
| **miRNAs** | **MedianKi-67 (0‑20)** | **Median Ki-67 ≥40** | **Fold change** | **T-test raw P‑value** | **T-test adj P‑value** | **AUC** |
| miR-1206 | 5.47 | 4.67 | 2.22 | 7.33E-05 | 0.05 | 0.26 |
| miR-886-5p | 6.05 | 5.33 | 2.04 | 8.64E-05 | 0.05 | 0.18 |

ER … estrogen receptor; Her2 … Her2 receptor status; adj … adjusted
